# Facile Synthesis of g-C_3_N_4_ Nanosheets/ZnO Nanocomposites with Enhanced Photocatalytic Activity in Reduction of Aqueous Chromium(VI) under Visible Light

**DOI:** 10.3390/nano6090173

**Published:** 2016-09-14

**Authors:** Xiaoya Yuan, Chao Zhou, Qiuye Jing, Qi Tang, Yuanhua Mu, An-ke Du

**Affiliations:** 1 College of Materials Science and Engineering, Chongqing Jiaotong University, Chongqing 400074, China; zczc6@126.com (C.Z.); jingqy1991@sina.com (Q.J.); tangqi99@163.com (Q.T.); myh25@cquc.edu.cn (Y.M.); 2 Chongqing Academy of Science and Technology, Chongqing 401123, China

**Keywords:** photoreduction, aqueous Chromium(VI), graphitic C_3_N_4_ nanosheets, ZnO, visible light

## Abstract

Graphitic-C_3_N_4_ nanosheets (CN)/ZnO photocatalysts (CN/ZnO) with different CN loadings were successfully prepared via a simple precipitation-calcination in the presence of exfoliated C_3_N_4_ nanosheets. Their morphology and structure were thoroughly characterized by powder X-ray diffraction (XRD), scanning electron microscopy (SEM), high-resolution transmission electron microscopy (HRTEM), X-ray photoelectron spectroscopy (XPS), UV-Vis diffuse reflectance spectroscopy (DRS) and photoluminescence spectra (PL). The results showed that hexagonal wurzite-phase ZnO nanoparticles were randomly distributed onto the CN nanosheets with a well-bonded interface between the two components in the CN/ZnO composites. The performance of the photocatalytic Cr(VI) reduction indicated that CN/ZnO exhibited better photocatalytic activity than pure ZnO under visible-light irradiation and the photocatalyst composite with a lower loading of CN sheets eventually displayed higher activity. The enhanced performance of CN/ZnO photocatalysts could be ascribed to the increased absorption of the visible light and the effective transfer and separation of the photogenerated charge carriers.

## 1. Introduction

Hexavalent chromium (Cr(VI)) is the most toxic of the chromium species and has been listed as one of the priority pollutants by the US Environmental Protection Agency (EPA) due to its notoriously toxic, mutagenic and carcinogenic activity to human beings [1,2]. Until now, a number of techniques have been developed for Cr(VI) elimination from waste water, such as chemical precipitation, reverse osmosis, ion exchange, foam flotation, electrolysis, adsorption, and photocatalytic reduction [3,4]. Among them, photocatalytic reduction is an efficient, active, and clean technology for Cr(VI) removal because of its acceptable cost and easy operation as well as the inexhaustible solar energy [5,6,7].

Among the oxide-based photocatalysts, ZnO has been widely studied for environmental remediation and is believed to be an alternative photocatalyst to TiO_2_ due to their similar band-gaps and similar photocatalytic mechanisms [8,9]. Some research about Cr(VI) photoreduction activity using ZnO photocatalysts has also been reported [10,11,12,13]. However, the high recombination of photoinduced electron-hole pairs, poor response to visible light and photocorrosion have hindered the application of ZnO in photocatalysis [14,15]. One of the effective strategies to improve the charge carrier separation and the visible-light harvesting of ZnO-based photocatalysts is to couple ZnO with narrow-band-gap semiconductors [16,17,18,19,20,21]. So far, a variety of semiconductors with narrow band-gaps including C_3_N_4_, Bi_2_O_3_, BiOI, V_2_O_5_, CdS, etc., have been used as sensitizers to design visible-light–driven ZnO-based composite photocatalysts. Coupling ZnO with the visible-light sensitizer not only enhances the light harvesting, but also facilitates charge separation in the photoexcited electron-hole pairs. The key to achieve the desired coupling effects is a good interface connection between two semiconductors with well-developed structures so that charge transfer can proceed smoothly across the interface [16,22].

Recently, graphite-like carbon nitride (g-C_3_N_4_), a metal-free layered polymer semiconductor, exhibited excellent photocatalytic activities toward many organic pollutants under visible-light irradiation and has attracted plenty of attention due to its appropriate band structure and visible-light adsorption ability as well as its excellent chemical stability and ease of large-scale preparation using various precursors [23,24,25,26,27,28]. Recent research about the exfoliated g-C_3_N_4_ shows that ultrathin g-C_3_N_4_ nanosheets (CN) do exhibit many superior properties to its bulk analogy [29,30,31,32,33], very similar to hotspot graphene [34,35]. Although there has been some work recently reported about the g-C_3_N_4_/ZnO composites [16,17,36,37,38,39,40,41], which is mainly focused on the photocatalytic oxidation property, to our knowledge, few reports using CN as the substrate to fabricate nanocatalysts for water purification and coupling ZnO with CN has rarely been reported so far for Cr(VI) photoreduction. In this study, CN/ZnO photocatalysts with different CN loadings were successfully prepared via a simple precipitation-calcination in the presence of exfoliated C_3_N_4_ nanosheets. The photocatalysts were thoroughly characterized by X-ray diffraction (XRD), scanning electron microscopy (SEM), high-resolution transmission electron microscopy (HRTEM), X-ray photoelectron spectroscopy (XPS), diffuse reflectance spectroscopy (DRS) and photoluminescence spectra (PL) and their visible-light photocatalytic properties were evaluated by Cr(VI) photoreduction. 

## 2. Results and Discussion

### 2.1. Charaterization of CN/ZnO Nanocomposites

**Figure 1:**
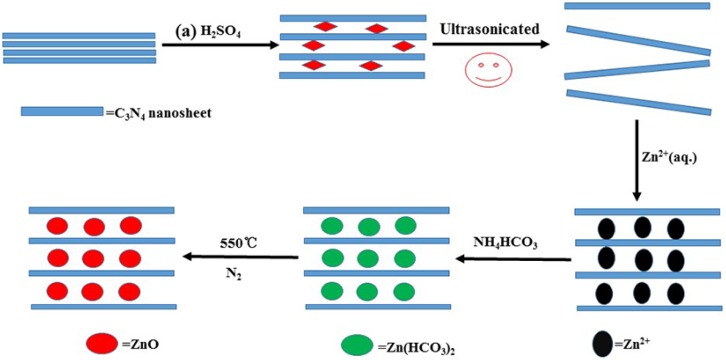
A schematic representation of the synthetic route to graphitic-C_3_N_4_ (g-C_3_N_4_) nanosheets (CN)/ZnO nanocomposites. CN: Graphitic-C_3_N_4_.

Figure 1 illustrates the fabrication process and formation mechanism for CN/ZnO nanocomposites. Firstly, H_2_SO_4_-C_3_N_4_ prepared via H_2_SO_4_ intercalation into bulk graphitic C_3_N_4_ was exfoliated into ultrathin CN nanosheets with few layers with the aid of ultrasonification, which was confirmed in previous studies [29,33]. As demonstrated in previous reports [23,25], CN sheets have triazine units on the basal planes and amino groups located at the edges. These functional groups act as anchor sites and Zn^2+^ could be adsorbed onto the surfaces of CN nanosheets due to the Van der Waals force and the coordination interaction between the metal ions and the functional groups. Secondly, the nuclei of the Zn(HCO_3_)_2_ precursor were formed in a short time upon the addition of NH_4_HCO_3_ [42] and gradually grew onto CN nanosheets at relatively high temperature. Finally, high-temperature treatment of CN/Zn(HCO_3_)_2_ under an N_2_ atmosphere gave the product CN/ZnO.

**Figure 2:**
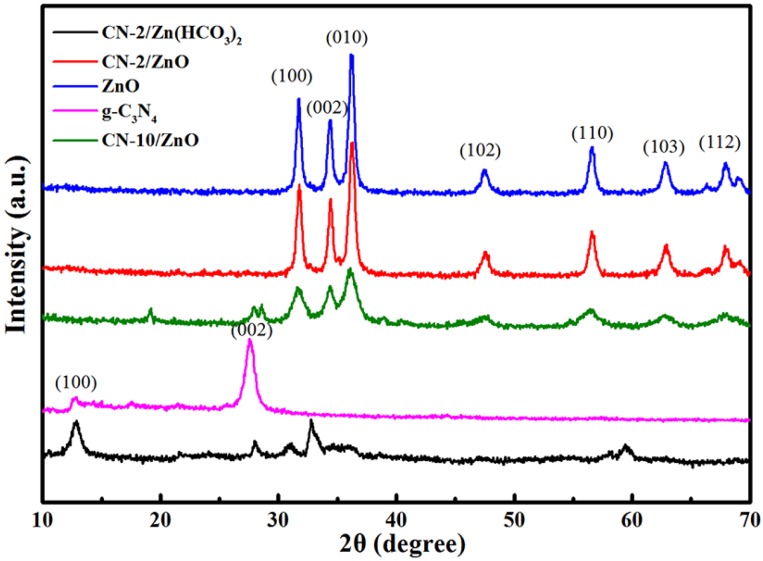
X-ray diffraction (XRD) patterns of ZnO, C_3_N_4_, CN-2/ZnO and CN-10/ZnO.

Figure 2 shows the XRD patterns of ZnO, C_3_N_4_ and CN-ZnO with different CN contents. For pure ZnO, the peaks at 31.7° (100), 34.4° (002), 36.2° (010), 47.3° (102), 56.5° (110), 62.7° (103) and 67.9° (112) matched well with the standard data of the ZnO hexagonal wurtzite phase (JCPDS No. 36-1451) [36] and no other ZnO crystallite phases in all the samples prepared herein were observed. Bulk g-C_3_N_4_ exhibited two peaks at 13.0° and 27.0° corresponding to the (100) and (002) crystal planes of g-C_3_N_4_, which were ascribed to the inter-planar stacking peak of aromatic systems and the in-plane structural packing motif, respectively [29,33]. No diffraction peaks of the crystalline g-C_3_N_4_ phase were detected in CN-2/ZnO with a lower content of CN, indicating CN nanosheets were totally exfoliated at lower loadings and uniformly dispersed in the CN-2/ZnO due to the effective prevention of the restacking of the CN layered by the ZnO particles, similar to other two dimensional (2D) lamellar material embedded in the composites [43,44,45]. With higher CN loadings, however, the as-prepared CN-10/ZnO samples exhibited a crystalline g-C_3_N_4_ peak, which was similar to recent reports [17]. Meanwhile, the ZnO crystal phase in the CN/ZnO composites was detected, which favored the photocatalytic properties of the as-prepared photocatalyst.

**Figure 3:**
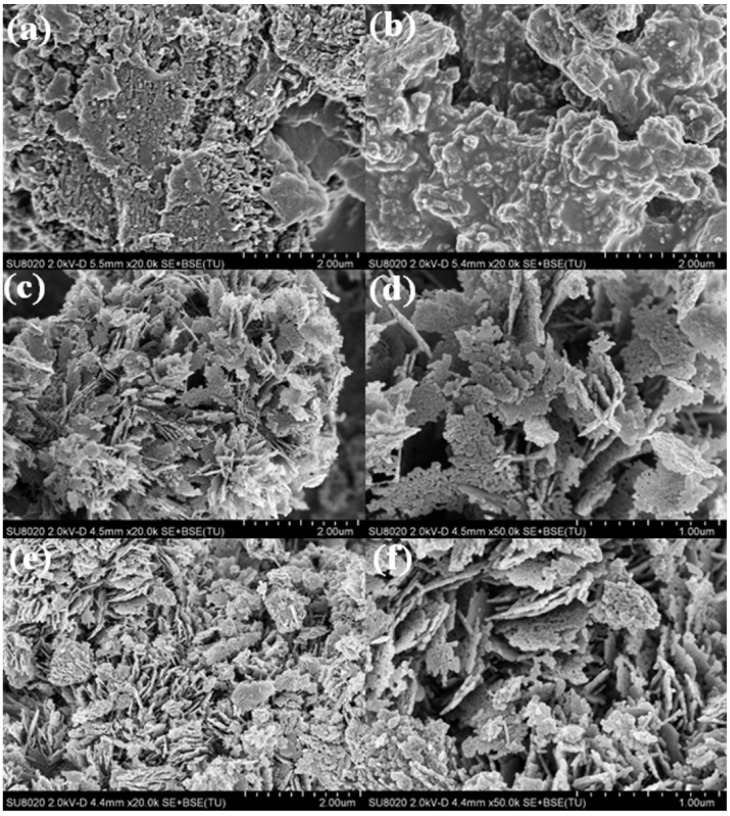
Scanning electron microscopy (SEM) images of (**a**) g-C_3_N_4_; (**b**) H_2_SO_4_-C_3_N_4_; (**c**,**d**) CN-2/ZnO; and (**e**,**f**) ZnO.

Figure 3 shows the typical SEM images of g-C_3_N_4_, H_2_SO_4_-C_3_N_4_, CN-2/ZnO and ZnO. g-C_3_N_4_ was composed of a large number of irregular aggregated particles about several micrometers in size and plenty of small pores resulting from gas discharged from the melamine decomposition could be detected in these particles (Figure 3a). After acid treatment, the H_2_SO_4_-C_3_N_4_ sample exhibited surface-smooth particles (Figure 3b). From Figure 3c,d, CN-2/ZnO exhibited an ordered sheet-like structure composed of aggregated spherical ZnO nanoparticles with a mean size of around 25 nm on the surface of the CN sheets, indicating that the CN sheets acted as the substrate for the formation of the ZnO precursor during the chemical precipitation process. Compared to the CN-2/ZnO nanocomposite, as seen from Figure 3e,f, pure ZnO displayed more disordered agglomerates composed of a larger diameter size (ca. 30–50 nm) and this phenomena was also found in graphene-based composites because the planar 2D material provided more substrates for the nuclei formation and the subsequent special confinement of the crystalline growth [46,47].

**Figure 4:**
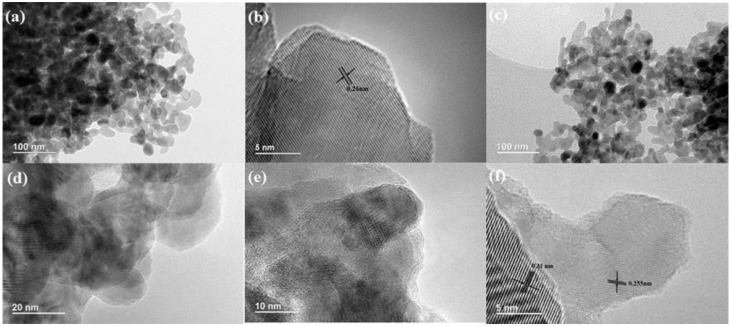
High-resolution transmission electron microscopy (HRTEM) images of (**a**,**b**) ZnO; and (**c**–**f**) CN-2/ZnO.

Figure 4 shows the typical HRTEM images of ZnO and CN-2/ZnO. The pure ZnO nanoparticles exhibited an irregular spherical morphology with a size diameter in the range of 20–100 nm (Figure 4a) and the lattice fringes were measured to be 0.26 nm (Figure 4b), which could be assigned to the (002) facets of the hexagonal wurtzite ZnO phase [36]. From Figure 4c–e, it was seen that ZnO nanoparticles were randomly and densely distributed on the surface of the CN sheets and showed a smaller particle size compared to that of pure ZnO. Two different kinds of lattice fringes were clearly exhibited (Figure 4f), one of *d* = 0.31 nm matching the (002) crystallographic plane of C_3_N_4_ [33], the other of *d* = 0.255 nm identical to the pure wurtzite ZnO nanoparticles as presented in Figure 4b, further indicating that the prepared sample consisted of CN and ZnO and the CN did not destroy the ZnO crystalline structure in our synthetic process. Moreover, the junction interface between the CN and ZnO phase in the composite was easily detected from Figure 4f, where the CN sheets acted as bridges for the connection between the ZnO nanoparticles, and consequently the CN sheets were densely decorated by the ZnO nanoparticles. The close connection between the two components was favorable for the increased separation of photogenerated carriers and would enhance the photocatalytic performance of the photocatalysts.

**Figure 5:**
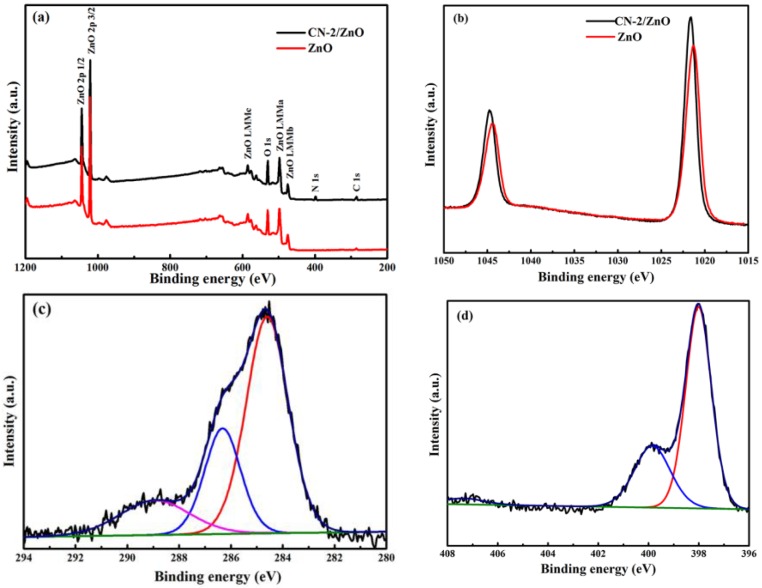
(**a**) X-ray photoelectron spectroscopy (XPS) survey spectra and (**b**) Zn2p peaks of CN-2/ZnO composite and ZnO; (**c**) high-resolution C1s and (**d**) N1s spectra for CN-2/ZnO composite.

Figure 5 provides the XPS survey spectra and Zn2p of the CN-2/ZnO composite and the ZnO as well as high-resolution C1s and N1s spectra for the CN-2/ZnO composite. The survey spectrum in Figure 5a clearly indicated that the composition of CN-2/ZnO included Zn, O, C and N elements. The peak located at 531.0 eV was associated with the O^2−^ ions in the ZnO [48]. The Zn2p spectrum of pure ZnO (Figure 5b) exhibits two peaks at 1021.2 eV and 1044.3 eV assigned to Zn2p_3/2_ and Zn2p_1/2_, respectively, which were consistent with the typical binding energy of Zn^2+^ in the ZnO [49,50]. However, the binding energies of Zn2p_3/2_ and Zn2p_1/2_ for the CN-2/ZnO composite were 1021.6 and 1044.8 eV, slightly higher than those for pure ZnO. Such a chemical shift of the Zn2p spectrum in the CN/ZnO composite could be ascribed to the formation of the N–Zn bonds [36,50]. The C1s spectra showed three deconvoluted peaks located at 284.6, 286.3 and 288.9 eV. The peak at 284.6 eV was exclusively assigned to surface adventitious carbon while the peaks at 286.3 and 288.9 eV were attributed to the sp^2^-hybridized carbon bonded to N inside the triazine rings and bonded to the –NH_2_ group, respectively [51]. The high-resolution N1s spectrum of CN-2/ZnO could be fitted into three different peaks. The main signals at 398.0 and 399.8 eV were assigned to the sp^2^-bonded N atoms in the triazine rings and the bridging N atoms in the N–(C)_3_ groups [29,51], while the weak peak associated with terminal amino functions exhibited much higher binding energy at 407.3 eV, probably due to the formation of the Zn–N bonds in the composite material [36,40,49].

**Figure 6:**
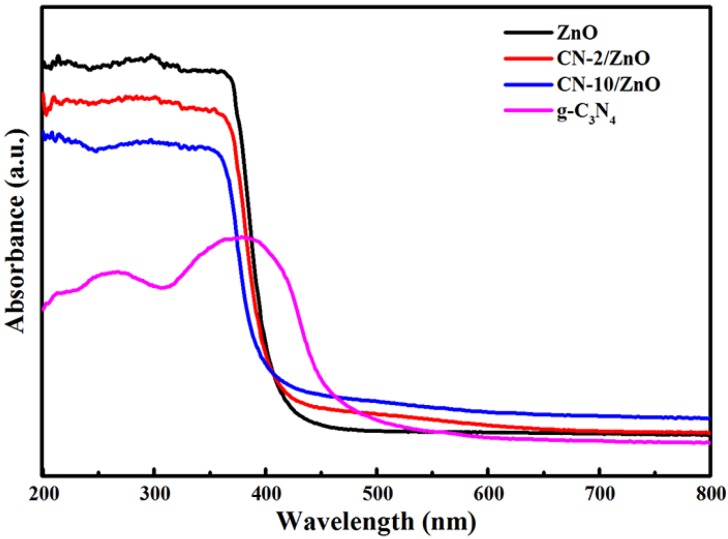
UV-Vis diffuse reflectance spectra of g-C_3_N_4_, ZnO, CN-2/ZnO and CN-10/ZnO photocatalysts.

The UV-Vis DRS spectra of g-C_3_N_4_, ZnO and different mass ratios of the CN/ZnO photocatalysts are shown in Figure 6. As expected, a sharp fundamental absorption edge for ZnO rose at ca. 400 nm, attributable to the 3.11 eV bandgap. The main absorption edge of the pure g-C_3_N_4_ occurred at ca. 470 nm, corresponding to a typical band-gap of 2.63 eV. Compared with pure ZnO, the absorption wavelength range of both CN-2/ZnO and CN-10/ZnO extended towards the visible-light region and the absorption intensity also increased, which demonstrated the effective surface coupling of ZnO with CN sheets and therefore an improved photocatalytic activity of the prepared photocatalysts could be expected under visible-light irradiation. The red-shifted absorption of the composites in the visible-light region was clearly enhanced with the increasing CN amount. These results implied that well-bonded interfaces between the CN nanosheets and ZnO phase could be formed [36,40] and thus make CN/ZnO photocatalysts shift to the lower energy region.

**Figure 7:**
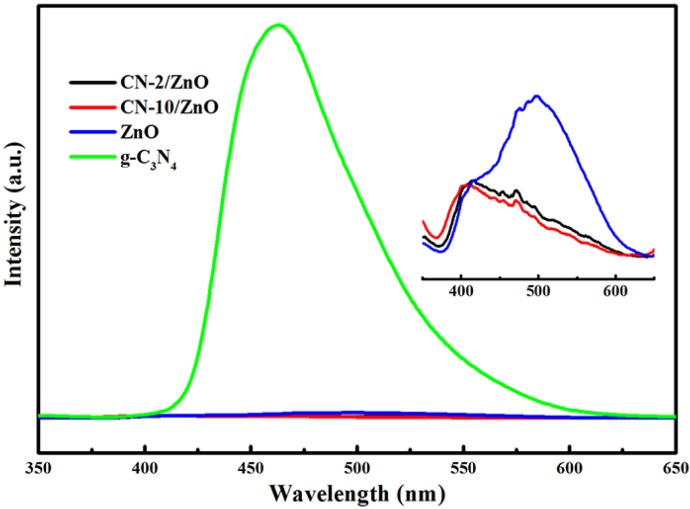
Photoluminescence spectra (PL) spectra of g-C_3_N_4_, ZnO and CN/ZnO photocatalysts. The inset shows the PL spectra of ZnO, CN-2/ZnO and CN-10/ZnO composites.

PL spectral analysis was conducted to demonstrate the migration, transfer, and recombination processes of photoinduced electron-hole pairs in the photoinduced system. Figure 7 shows PL spectra of the g-C_3_N_4_, ZnO and CN/ZnO photocatalysts. A main strong emission peak at 498 nm and a shoulder-peak at around 417 nm for pure ZnO were observed, which was consistent with other reports [40,52]. The peak at 463 nm of the bulk g-C_3_N_4_ was almost not detected in the CN/ZnO composites due to the low content of CN. Compared to pure ZnO, the PL peak intensity at 498 nm for both CN/ZnO photocatalysts was significantly reduced, indicating that the modification of ZnO with few-layered CN nanosheets could effectively suppress the recombination of photoinduced electron-hole pairs in the composites and thus efficiently separate the photoinduced electrons in electron-transfer processes [53,54]. Combined with the enhanced absorption in the visible-light region presented in the UV-DRS analysis, therefore, the incorporation of CN nanosheets into ZnO was greatly expected to enhance the photocatalytic performance of the CN/ZnO composites under visible-light irradiation.

### 2.2. Photocatalytic Reduction of Aqueous Chromium(VI) under Visible Light

**Figure 8:**
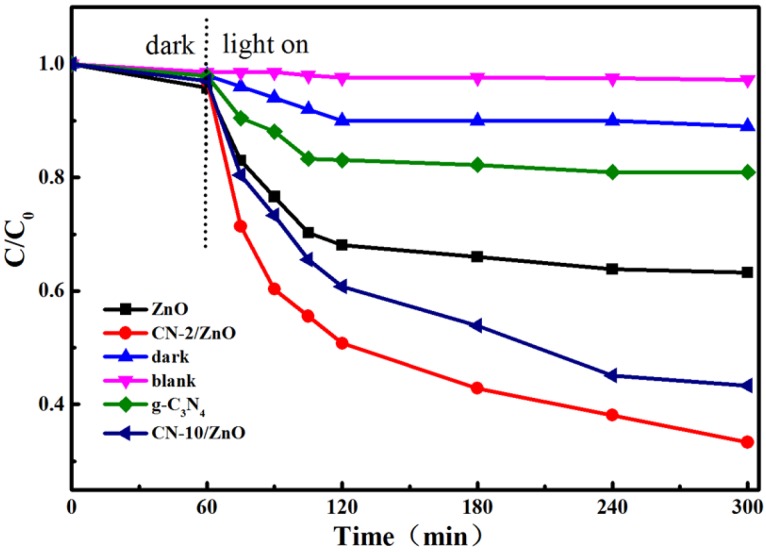
Photoreduction of aqueous Cr(VI) over g-C_3_N_4_, ZnO and CN/ZnO photocatalysts under visible light. C_0_ and C are the initial concentration and solution concentration of scheduled irradiation intervals.

Figure 8 presents the photocatalytic reduction of aqueous Cr(VI) over the g-C_3_N_4_, ZnO and CN/ZnO photocatalysts under visible light. The control experiments indicated that aqueous Cr(VI) was very stable under visible light (blank experiments) and the adsorption removal ratio of aqueous Cr(VI) was negligible (about 4%) for the CN-ZnO composites (dark experiments). The photoreduction rate of aqueous Cr(VI) was 18% and 34% for pure g-C_3_N_4_ and ZnO under 240 min irradiation. However, after some CN nanosheets were incorporated into the ZnO, the reduction rate of the Cr(VI) was increased to 70% and 47% for CN-2/ZnO and CN-10/ZnO, strongly suggesting that the modification with CN nanosheets could greatly enhance the photocatalytic reduction activity of CN/ZnO composites. Compared to other C_3_N_4_- [55,56] and ZnO-based photocatalysts for visible-light-driven Cr(VI) photoreduction [42,57], the CN/ZnO prepared herein showed enhanced photoreduction activity, indicating that modification with CN was effective for improving the photo-activity of ZnO. Furthermore, the lower loading of the CN content for CN-2/ZnO exhibited better activity than that for CN-10/ZnO and this was because the excess CN nanosheets may act as a recombination center, covering the active sites on the surface of ZnO particles and therefore reducing the efficiency of charge separation.

**Figure 9:**
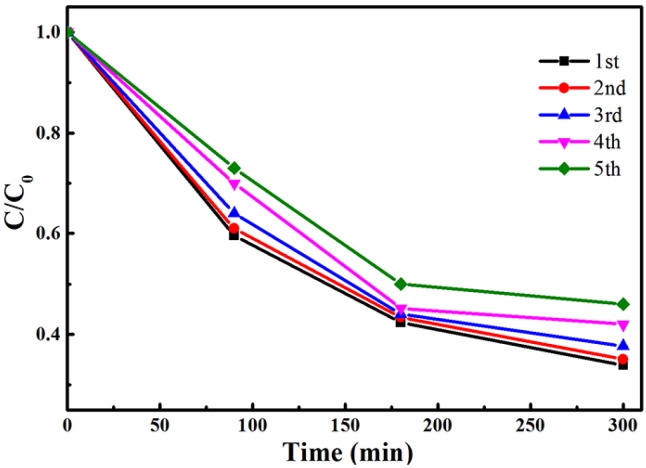
Cycling runs for the photoreduction of Cr(VI) by CN-2/ZnO composite under visible light.

The photostability of the CN-2/ZnO in the circulating runs for the photocatalytic reduction of Cr(VI) under visible-light irradiation was further examined (Figure 9). After 240 min of irradiation in each cycle, the photocatalyst was separated from the aqueous suspension by centrifugation and washed with deionized (DI) water. As shown in Figure 9, there was no significant decrease of the photocatalytic activity after five cycles. Compared to the recycled property reported by Wang [17], where bulk g-C_3_N_4_/ZnO showed dramatically reduced activity in the first cycle of Cr(VI) photoreduction, apparently the CN/ZnO composite had good photostability and chemical stability under the studied conditions, indicating that the photocorrosion effect of ZnO was effectively suppressed by CN hybridization [14,58]. The improved photoreduction activity could be ascribed to the increased absorption in the visible-light range and the enhanced charge separation efficiency at the interface of ZnO and CN nanosheets [16,36]. Furthermore, compared to those reports where better activities were obtained with the use of additional scavengers [11,55,57,59], this method described herein will be highly desirable for environmental remediation due to no additional scavengers in this process.

**Figure 10:**
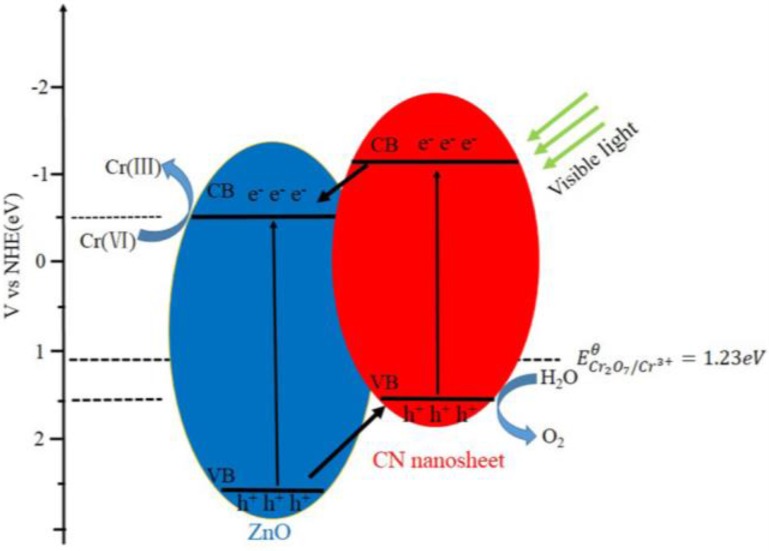
Schematic illustration of the mechanism of electron-hole separation and transport and photocatalytic activity of CN/ZnO photocatalyst under visible-light irradiation.

On the basis of the above results presented in this work, a synergistic mechanism between ZnO and CN nanosheets for the improved photoreduction of Cr(VI) is proposed as illustrated in Figure 10. Under visible-light irradiation, both CN sheets and ZnO can be excited and produce photogenerated electron-hole pairs. Since the conduction band (CB) edge of C_3_N_4_ (−1.12 eV) [60] is more negative than that of ZnO (−0.5 eV) [58], the photogenerated electrons on the surface of CN sheets can readily transfer to the CB of ZnO via the well-developed interface and thus reduce ${\mathrm{Cr}}_{2}O_{7}^{2-}$ to Cr^3+^ on the surface of the ZnO particles and the holes oxidize water to form O_2_, as illustrated in the following equations [61]. As depicted in Figure 10, this transfer favors electron-hole separation and leads to large numbers of electrons on the ZnO surface and holes on the g-C_3_N_4_ surface, respectively, thus promoting the photocatalytic reduction of aqueous Cr(VI).

Cr_2_O_7_^2−^ + 14H^+^ + 6e → 2Cr^3+^ + 7H_2_O (1)

2H_2_O + 4h^+^ → O_2_ + 4H^+^ (2)

## 3. Materials and Methods

### 3.1. Preparation of H_2_SO_4_-Intercalated C_3_N_4_ (H_2_SO_4_-C_3_N_4_)

All chemicals were used as received with any purification. The bulk g-C_3_N_4_ was prepared by direct pyrolysis thermal polycondensation of melamine in the semi-closed system. In a typical synthesis, 10 g of melamine was placed in an alumina crucible with a cover and then heated to 550 °C with a heating rate of 3 °C·min^−1^, and kept at this temperature for 4 h in static air.

The grey-white H_2_SO_4_-C_3_N_4_ was obtained by stirring bulk g-C_3_N_4_ powder in concentrated H_2_SO_4_ (98%) at room temperature as described in our previous report [33]. Briefly, the as-prepared yellow C_3_N_4_ powder (1.0 g) was mixed with concentrated H_2_SO_4_ (10 mL) and stirred for 8 h at room temperature for its intercalation, during which the solution color changed from yellow to grey. Then the mixture was slowly poured into deionized water (200 mL). The obtained light-grey suspension was filtrated, washed repeatedly with DI water to remove the residual acid and finally freeze-dried under vacuum to give the grey-white H_2_SO_4_-C_3_N_4_. The H_2_SO_4_ content in this sample was estimated to 30% by thermal analysis.

### 3.2. Preparation of CN/ZnO Nanocomposites

CN/ZnO nanocomposites with different theoretical content of C_3_N_4_ (2% and 10%) and pure ZnO nanoparticles used in this work were prepared by a reaction similar to that described by Pan [62]. In brief, H_2_SO_4_-C_3_N_4_ (60 mg) was ultrasonicated in DI water (100 mL) for 5 h to give homogeneous colloidal suspension containing large amount of ultrathin few-layered CN nanosheets [29,33]. Then ZnSO_4_·7H_2_O (6.9 g) was added and ultrasonicated for another 30 min to ensure the uniform adsorption of Zn^2+^ onto the surface of CN nanosheets. Aqueous solution (100 mL) containing NH_4_HCO_3_ (2.9 g) was added dropwise into the above solution at 60 °C within 10 min and this mixture solution was kept stirring at this temperature for 3 h, during which a lot of pungent NH_3_ was produced. The white precipitate (CN/Zn(HCO_3_)_2_) was collected by filtration, washed for three times with distilled water and then dried in vacuum oven at 60 °C for 24 h. Finally, this product was calcined at 550 °C for 2 h under N_2_ atmosphere to obtain the grew-yellow powder CN/ZnO nanocomposites with 2% theoretical content of CN. The as-synthesized CN/ZnO samples with 2.0% and 10% theoretical CN content were named as CN-2/ZnO and CN-10/ZnO. For comparison, pure ZnO was prepared with the same procedure without the use of H_2_SO_4_-C_3_N_4_.

### 3.3. Characterization

SEM images were obtained with a Hitachi SU8020 scanning electron microscope (Hitachi Ltd., Tokyo, Japan) with acceleration voltage of 20 kV. HRTEM was performed on a FEI Tecnai G2 F20 field-emission transmission electron microscopy (Hillsboro, OR, USA) at an accelerating voltage of 200 kV. XRD were recorded on a PANalytical X’pert Pro powder diffractometer (Almelo, The Netherland) with Cu-Ka radiation (λ = 1.5418 Å) with a scan step of 0.013°. XPS measurements were performed on a Thermo Fisher ESCALAB 250Xi photoelectron spectrometer (Waltham, MA, USA) using monochromatic Al Kα X-ray source (*hv* = 1486.6 eV). DRS was carried out on a Shimadzu UV-2450 UV-Vis spectrophotometer (Kyoto, Japan) using BaSO_4_ as the reference sample. PL was measured with a Hitachi F-7000 fluorescence spectrophotometer (Tokyo, Japan) using a Xe lamp as excitation source with optical filters.

### 3.4. Photocatalytic Tests

The photocatalytic reduction of aqueous Cr(VI) were carried out in a homemade photochemical reactor under visible-light irradiation. The visible-light source was provided by a 500 W Xe lamp with maximum wavelength emission at 470 nm (Shanghai Jiguang Special Lighting Factory, Shanghai, China). The lamp source was laid in the empty chamber of the annular quartz condensing tube with a circulating water jacket outside to immediately remove the heat released from the lamp. The distance between the light source and the tube containing the reaction mixture was set to be 15 cm. In each experiment, the photocatalyst (50 mg) was dispersed in 50 mL aqueous Cr(VI) solutions of different concentrations. Prior to irradiation, the mixture solution was magnetically stirred for 60 min in the dark to establish the adsorption-desorption equilibrium. During the irradiation, 4 mL of the reaction solution was withdrawn at certain time intervals and centrifuged to remove the photocatalyst from the solution. The Cr(VI) content in the supernatant solution was determined spectrophotometrically at 540 nm using the diphenylcarbazide method [41] (Evolution 600 UV-Vis spectrophotometer, Thermo Fisher Scientific Inc., Waltham, MA, USA).

## 4. Conclusions

In summary, CN/ZnO photocatalysts were successfully prepared via a facile precipitation-calcination in the presence of exfoliated C_3_N_4_ nanosheets. A well-bonded interface was formed between ZnO and CN nanosheets in the as-prepared photocatalysts. CN/ZnO exhibited better photocatalytic Cr(VI) reduction activity than pure ZnO under visible-light irradiation. The enhanced photoreduction performance of the CN/ZnO photocatalyst was be ascribed to the increased absorption of the visible light and the effective transfer and separation of the photogenerated charge carriers at the interface. The CN/ZnO photocatalyst prepared herein is being further evaluated by removing other pollutants and has great potential in water remediation.
